# Inborn errors of immunity and related microbiome

**DOI:** 10.3389/fimmu.2022.982772

**Published:** 2022-09-13

**Authors:** Raja Hazime, Fatima-Ezzohra Eddehbi, Saad El Mojadili, Nadia Lakhouaja, Ikram Souli, Abdelmouïne Salami, Bouchra M’Raouni, Imane Brahim, Mohamed Oujidi, Morad Guennouni, Ahmed Aziz Bousfiha, Brahim Admou

**Affiliations:** ^1^ Laboratory of Immunology, Center of Clinical Research, Mohammed VI University Hospital, Marrakech, Morocco; ^2^ Biosciences Research Laboratory, Faculty of Medicine and Pharmacy, Cadi Ayyad University, Marrakech, Morocco; ^3^ Pediatric infectious and Immunology Department, Ibn Rochd University Hospital, Casablanca, Morocco; ^4^ Laboratory of Clinical Immunology inflammation and Allergy, Faculty of Medicine and Pharmacy, Hassan II University, Casablanca, Morocco

**Keywords:** inborn errors of immunity, microbiome, dysbiosis, diagnostic strategy, therapeutic strategies

## Abstract

Inborn errors of immunity (IEI) are characterized by diverse clinical manifestations that are dominated by atypical, recurrent, chronic, or severe infectious or non-infectious features, including autoimmunity, lymphoproliferative disease, granulomas, and/or malignancy, which contribute substantially to morbidity and mortality. Some data suggest a correlation between clinical manifestations of IEI and altered gut microbiota. Many IEI display microbial dysbiosis resulting from the proliferation of pro-inflammatory bacteria or a decrease in anti-inflammatory bacteria with variations in the composition and function of numerous microbiota. Dysbiosis is considered more established, mainly within common variable immunodeficiency, selective immunoglobulin A deficiency, severe combined immunodeficiency diseases, Wiskott–Aldrich syndrome, Hyper-IgE syndrome, autoimmune polyendocrinopathy–candidiasis–ectodermal-dystrophy (APECED), immune dysregulation, polyendocrinopathy, enteropathy X-linked (IPEX) syndrome, IL-10 receptor deficiency, chronic granulomatous disease, and Kostmann disease. For certain IEIs, the specific predominance of gastrointestinal, respiratory, and cutaneous involvement, which is frequently associated with dysbiosis, justifies the interest for microbiome identification. With the better understanding of the relationship between gut microbiota, host immunity, and infectious diseases, the integration of microbiota modulation as a therapeutic approach or a preventive measure of infection becomes increasingly relevant. Thus, a promising strategy is to develop optimized prebiotics, probiotics, postbiotics, and fecal microbial transplantation to rebalance the intestinal microbiota and thereby attenuate the disease activity of many IEIs.

## 1 Introduction

Inborn errors of immunity (IEI) comprise 485 disorders with at least 430 known gene defects (1–3). Owing to the large number of manifestations that are more related to immune system dysregulation than its deficiency, the denomination “primary immune deficiencies” is too restrictive; therefore, the term “inborn errors of immunity’’ should be used instead. Atypical, recurrent, chronic, or severe infections dominate the clinical manifestations of IEI, which may also be associated with non-infectious complications including autoimmunity, lymphoproliferative disease, granulomas, and/or malignancy, thereby contributing considerably to morbidity and mortality (4). Previous studies suggest a correlation between clinical manifestations of IEI, including common variable immunodeficiency (CVID) and IPEX, and altered gut microbiota (5–7). These commensal microorganisms correspond to various microbes that constantly interact with the host at several sites, including skin and mucosal surfaces such as gastrointestinal and respiratory tracts. Interestingly, IEIs provide rare opportunities to assess the influence of deficient immunity on human microbiome and, in turn, how the compromised microbiome may interplay with the host to induce pathology. A microbiota comprises trillions of microbes that continuously interact with the host at several sites, such as the skin and mucosal surfaces, including the gut and respiratory tract. A microbiome includes a community of live microorganisms (bacteria, viruses, fungi, and protozoa), the microbial structural elements, metabolites, and their ecosystem (8). Therefore, it is predictable that these commensal microorganisms play a key role in numerous host functions including immunity (9). Microbial dysbiosis is mainly caused by genetic predisposition, in addition to its well-known causative factors such as infections and changes in diet and nutritional status and the use of antibiotics, gastric acid suppressants, and anticancer drugs (10).

In this review, we aimed to highlight the existing knowledge on the interactions between the microbiota and the host immune system in IEI, focusing mainly on the molecular mechanisms of gut microbiota–host interactions and the disruption of these mechanisms in specific forms of IEI.

## 2 Interplay between the gut microbiome and IEI

Gut microbiota is currently considered an important factor in maintaining cellular homeostasis (8). The microbiota comprises over 500 different species (11) providing metabolic functions, preventing colonization by pathogens, and promoting immune function. The host immune system and the gut microbiota interact symbiotically, engaging innate and adaptive host immune responses, such as mucus secretion, antimicrobial proteins (AMPs), and immunoglobulin A (IgA) production (10). Healthy intestinal microbiota generally comprises two major phyla *Firmicutes* and *Bacteroidetes*, combined with *Actinobacteria*, *Proteobacteria*, *Fusobacteria*, and *Verrucomicrobia* (12). *Firmicutes* are gram-positive bacteria, which are dominated by the class *Clostridia* and are associated with *Lactobacillaceae, Enterococcaceae*, and *Lactococcus* spp., whereas *Bacteroidetes* are gram-negative bacteria, including *Bacteroides thetaiotaomicron*, *Bacteroides fragilis*, and *Bacteroides ovatus* (13). Gut microbiota also includes fungi (*Candida, Saccharomyces, Malassezia*, and *Cladosporium)* (14), protozoa, and viruses (15).

In genetically predisposed individuals, imbalances in microbiota–immunity interactions under certain environmental factors appear to contribute to the pathogenesis of immune mediated disorders (16, 17). In IEI diseases, which are mostly monogenic disorders and deficiencies in the adaptive or innate immune system lead to an abnormal inflammatory response, damage of the gastrointestinal tract, and an increased risk of developing inflammatory and autoimmune disorders (18). For instance, in CVID, the disruption of the gut barrier due to recurrent infections (19) and the reduction of secretory IgA (3) increase microbial translocation (20), in addition to lipopolysaccharide (LPS) permeability (21–23). LPS activates toll-like receptor 4 (TLR4) on innate immune cells such as macrophages, neutrophils, and mast cells to release pro-inflammatory mediators and reactive oxygen species (ROS) (24).

### 2.1 Severe combined immunodeficiencies

Severe combined immunodeficiencies (SCIDs) are a group of monogenic disorders defined by profound CD3 T-lymphocyte depletion (25) associated for some with profound B-lymphopenia (1). To date, 19 molecular defects have been reported as the causes of these deficits: JAK3, IL2RG, RAG1, RAG2, IL7R, DCLRE1C, PRKDC, CD3D, CD3E, CD3Z, LIG4, PTPRC, LAT, CORO1A, FOXN1, ADA, AK2, RAC2, and NHEJ1 (2). SCIDs are characterized by the early onset of severe infections and results in premature death in the absence of early management based on hematopoietic stem cell transplantation (HSCT) (26), gene therapy (27), or enzyme replacement therapy (28) depending on the SCID type. HSCT can generate potentially lethal complications such as graft-versus-host disease (GvHD), the occurrence of which may be influenced by several factors including the gut microbiota (29). In a pilot study, Lane et al. examined gut microbiota before and after HSCT in patients with SCID; two patients were IL2RG-deficient and one patient was RAG1-deficient. Despite the small number of patients analyzed, the study demonstrated that bacterial taxonomy changed over time, producing distinct pre- and post-HSCT microbiota populations with low microbial diversity associated with the dominance of varying species, mainly *Escherichia*, *Staphylococcus*, and *Enterococcus* (30, 31). Clarke et al. analyzed gut microbiota and immune system changes in patients with X-linked SCID (mutations of *Il2RG*) treated by gene therapy. In patients with a restored immune system, normalization of the gut microbiota with a progressive normalization of the T-cell receptor (TCR) repertoire was observed (32).

These findings highlight the importance of identifying bacterial species based on their beneficial or pathogenic effects to identify new biomarkers for monitoring intestinal inflammation during HSCT. Furthermore, in patients with SCID, microbiota present in low concentrations may present opportunities for therapeutic targets, making specific fecal microbiota transplantation (FMT) a potential therapy for intestinal diseases (18).

### 2.2 Wiskott–Aldrich syndrome

Wiskott–Aldrich syndrome (WAS) is an X-linked IEI classified as a combined immunodeficiency (CID) associated with congenital thrombocytopenia (2). WAS is characterized by several clinical disorders, such as recurrent infections, eczema, and an enhanced incidence of both autoimmunity and malignancies (33). The syndrome is caused by mutations in the *WAS* gene that encodes the WAS protein (WASp), which is involved in relaying signals from the cell surface to the actin cytoskeleton and is expressed exclusively in hematopoietic cells (34). Up to 5–10% of the patients with WAS develop inflammatory bowel disease (IBD), especially the early-onset and severe IBD (35, 36). IBD is classically defined as a disorder with chronic idiopathic and recurrent inflammation of the gastrointestinal tract (37), which results from the dysbiosis of the intestinal microbiota associated with a deficient host immune system. To better understand the role of microbial dysbiosis in IBD in patients with WAS, Zhang et al. studied commensal microorganisms in children with WAS and those in WASP-deficient mice. They reported a lower abundance of *Bacteroidetes* and *Verrucomicrobia* in children with WAS while *Proteobacteria* was markedly higher compared to healthy controls. Furthermore, among the children with WAS, those with IBD exhibited more severe microbial dysbiosis (38). Another feature of patients with WAS is oral involvement, especially gingivitis and periodontitis, with premature loss of deciduous and permanent teeth. In this context, by focusing on the oral microbiota in patients with WAS, Lucchese et al. demonstrated that *Fusobacterium nucleatum* (most prevalent periodontal bacteria) together with P*orphyromonas gingivalis* and *Tannerella forsythia* contributed to the periodontitis onset (39). These bacterial species are the physiological components of the oral microbiota. However, in the context of immune deficiency, notably that of WASp, these bacterial species can proliferate and become pathogenic, leading to periodontal lesions (39).

Owing to the rarity of WAS, studies on microbiota disorders have been conducted in small cohorts with statistically non-significant results, which require confirmation in a larger sample size of the population.

### 2.3 Common variable immunodeficiency

CVID is the most frequent IEI in adulthood with a prevalence of approximately one per 25,000 people (40). CVID is characterized by a decrease in the serum IgG concentrations (at least 2 SD under the mean for age), IgA, and/or IgM and altered antibody synthesis in response to pathogens and vaccines (41). The clinical features are mainly recurrent respiratory infections caused by extracellular encapsulated bacteria, digestive features, and many other disorders (41). Recent studies suggest that the complications observed in CVID may be induced by immune dysregulation owing to both altered microbiota composition and increased microbial translocation (24). Additionally, microbial dysbiosis has also been suggested to play a role in inflammatory and immune dysregulation in CVID *via* epigenetic mechanisms (42).

Jørgensen et al. were the first to investigate the microbiota in patients with CVID (21). They performed a 16S ribosomal RNA analysis on fecal samples from 44 patients with CVID, 45 patients with IBD, and 263 healthy controls, and combined these results with the plasma measurements of LPS (endotoxemia marker), soluble sCD14 (monocyte activation marker), and sCD25 (T-cell activation marker) in an expanded sample of 104 patients with CVID and 30 healthy controls to assess the level of systemic immune activation in CVID. They determined a lower alpha diversity in patients with CVID compared with healthy controls, as well as low levels of the genus *Bifidobacterium* in patients with CVID (21), which is suggested to restore healthy properties and is used in probiotic supplements (43). Conversely, *Clostridia*, *Bacilli*, and *Gammaproteobacteria* species were markedly more abundant in the CVID group, inferring that these are considerably associated with CVID. Only the CVID “Complications” subgroup was affected by the reduction in alpha diversity and the increase in LPS levels, whereas the “Infection only” subgroup had similar alpha diversity and LPS levels compared to those of controls (21). Increased microbial translocation of gram-negative bacteria is indicated by the presence of LPS in the systemic circulation (42). Additionally, there were increased serum endotoxin levels and reduced IgA levels of IgA, and no correlation was detected for IgG and IgM levels (21). Shulzhenko et al. reported that in patients with CVID, mucosal IgA levels were lower in patients with enteropathy compared to those without the disease. Furthermore, they identified three different bacterial taxa that may contribute to CVID enteropathy, namely, *Acinetobacterbaumannii*, *Geobacillus*, and *otu_15570* bacterium (3). *Acinetobacterbaumannii* appeared to be the most involved in enteropathy occurrence during CVID. *Geobacillus* is also a well-known pro-inflammatory cytokine inducer in the small intestine of mice and has some genetic properties identical to segmented filamentous bacteria (SFB) (44).

Macpherson et al. (45) hypothesized that dietary metabolites could explain the relationship between altered gut microbiota and systemic inflammation in CVID. They noted that all patients with CVID had increased plasma concentrations of trimethylamine N-oxide (TMAO), which is related to systemic inflammation (elevation of TNFα and IL-12 levels), and higher stool abundance of *Gammaproteobacteria* (45). These findings suggest that TMAO may be the link between disturbed gut microbiota and systemic inflammation in patients with CVID (45). This relationship with certain commensal bacteria supports the idea that microbiota may be a potential treatment target to minimize TMAO production and thereby, systemic inflammation in CVID.

Conversely, fatty acid (FA) disorders have also been linked to gut microbial dysbiosis during various inflammatory disorders. A recent study on the plasma FA composition in patients with CVID demonstrated that these patients had an altered FA profile with lower proportions of eicosapentaenoic and docosahexaenoic FAs and a decreased anti-inflammatory index (46). Furthermore, a favorable FA profile was correlated with serum IgG levels, confirming the relationship between these FAs and IgG, which is an important but mostly correctable immunological parameter of CVID (46).

### 2.4 Selective IgA deficiency

As an important factor of mucosal immunity, IgA levels in the intestinal lumen are involved in maintaining diverse and balanced microbiome. IgA confers immunological tolerance to commensal bacteria (47) and promotes expulsion of the pathogenic bacteria and toxins from the gut epithelia (48), thereby maintaining gut barrier integrity (42). Furthermore, secretory IgA (sIgA) is capable of specifically adhering to and being translocated by M cell Peyer’s patches (PP) in mouse and human intestines, allowing for antigen sampling by dendritic cells (DCs) under conditions of neutralization, which is essential to the homeostasis of the mucous surfaces (49, 50). However, this function is not conferred by IgA antibodies as they cannot adhere to M cells (51). There are two pathways of IgA production by gut plasma cells—T-cell-dependent and T-cell-independent—which require cooperation with epithelial cells, DCs, and innate lymphoid cells (ILCs) (10). The first pathway typically occurs in Peyer’s patches and microbiota-specific, whereas the second pathway mostly takes place in the lamina propria and isolated lymphoid follicles, leading to the exclusion of microorganisms from the gut (52). Additionally, the microbiota induces IgA2 class switching through a CD4+ T-cell-independent pathway by linking B cells in lamina propria to intestinal epithelial cells *via* a TLR-inducible and APRIL-requiring signaling program (53). Notably, IgA1 is less resistant to bacterial proteases than IgA2. Interestingly, some species of the microbiota, such as *Sutterella*, degrade both IgA and its secretory constituent, a peptide involved in the stability of IgA in the lumen. Therefore, those microbiota members are inversely correlated to the level of IgA in feces (54). In both mice and humans, IgA predominantly targets the commensal bacteria in the small intestine (55), which may explain why alterations of the gut microbiota were observed in the small intestine, while the large intestine communities were much less affected by the absence of IgA (56).

Selective IgA deficiency is the most prevalent primary immunodeficiency (18) and is associated with recurrent mucosal infections, mostly due to bacteria (e.g., *Haemophilus influenzae* and *Streptococcus pneumoniae*), atopy, and autoimmunity (e.g., celiac disease).

To investigate the overall impact of digestive IgA deficiency on the gut microbiome, Fadlallah et al. (57) using a metagenomic approach to explore microbiota in specific IgA-deficient patients demonstrated that selective IgA deficiency in humans was not associated with large quantitative disturbances in the gut microbial ecosystem. Instead, it was characterized by the expansion of pro-inflammatory bacteria, reduction in anti-inflammatory commensals, and disruption of the “obligatory” bacterial network (57). The same finding was reported by Moll et al., who concluded that the microbiota of IgA-deficienct patients was enriched in species with increased pro-inflammatory potential (58).

Furthermore, selective IgA deficit is characterized by a mild phenotype, which may be explained by the partially compensation of its deficiency *via* IgM secretion (59). In IgA-deficient mice, the serum IgG responses, reflecting a systemic immune response, were spontaneously directed against commensal bacteria (60).

### 2.5 Hyper-IgE syndrome

Hyper-IgE syndromes (HIESs) are a combined immunodeficiency characterized by highly elevated serum IgE levels (2). To date, several mutations are involved in the occurrence of HIES: the most common one is the dominant-negative mutation in the human signal transducer and activator of transcription 3 (*STAT3*) gene (61, 62). HIESs are associated with recurrent staphylococcal skin abscesses, chronic mucocutaneous candidiasis (CMC), cyst-forming pneumonia, and skeletal abnormalities (63). Additionally, STAT3 plays a crucial role in signal transduction triggered by numerous cytokines, including IL-6, IL-10, IL-11, IL-17, IL-21, and IL-22, thereby facilitating the differentiation of naive CD4+T cells into Th17 cells, neutrophil proliferation, and chemotaxis (64). This may explain the defect of Th17 cell differentiation in patients with STAT3 mutations (65), which results in increased susceptibility to candida infection (66). Furthermore, IL-17 and IL-22 play a role in the upregulation of antimicrobial peptides involved in staphylococcal atopic dermatitis and CMC (67).

To evaluate the effect of STAT3 deficiency on the oral microbiome, Abusleme et al. (68) investigated fungal and bacterial communities in autosomic dominant HIES compared to healthy volunteers (68). In actively infected patients, severe fungal dysbiosis with the predominance of *Candida albicans* and minimal prevalence of health-associated fungi *(C. parapsilosis, Boletus*, *and Penicillium*) was reported. Thus, these data support the essential role of the STAT3/Th17 axis in maintaining *C. albicans* as a commensal organism. Oral bacterial communities were also dysbiotic in AD-HIES, in which the presence of active oral candidiasis was associated with increased prevalence of *Streptococcus mutans* and *Streptococcus oralis* in the oral mucosa (68). This association supports the existence of a synergistic relationship between *C. albicans* and oral *Streptococci* (69). Previous studies using mouse models have demonstrated that *S. oralis* colonization of the oral and gastrointestinal tracts increased in the presence of *C. albicans* (70).

### 2.6 IEI with immune dysregulation

Single-gene IEIs generally refer to monogenic autoimmune disorders, such as immune dysregulation, polyendocrinopathy, and enteropathy with X-linked inheritance (or IPEX syndrome) caused by mutations in the forkhead box P3 (Foxp3), and APECED caused by mutations in the autoimmune regulatory transcription factor (AIRE) (71). Previous studies concluded that single-gene IEIs were not influenced by commensal microbial regulation since they developed even in germ-free (GF) mice (72, 73). However, new evidence reveals that while microbiota has no impact on the disease onset; the severity of many single-gene IEIs varies with the microbiome (71).

#### 2.6.1 IPEX syndrome

FOXP3 is an important transcription factor that plays a central role in the development and functioning of CD4+ regulatory T cells (Tregs) (74, 75), which restricts immune activation and is essential to prevent systemic autoimmunity. Most gut Treg cells are derived from conventional FOXP3neg CD4+ T-cells to generate tolerogenic responses to microbiota and food antigens (76). Besides, microbiota may also produce immunomodulatory metabolites through the fermentation of indigestible fibers, such as short-chain fatty acids (SCFAs) including butyrate, which can modulate the immune system by inducing extrathymic generation of Treg cells (77). Mutations in FOXP3 lead to IPEX syndrome, which should be considered in males with the following clinical signs: early-onset IBD, endocrinopathy (most commonly type 1 diabetes mellitus and autoimmune thyroid disease), and dermatitis (most commonly eczema) (78). IPEX syndrome is associated with decreased diversity in gut microbiota, as reported by studies on the microbiota of mice and patients with IPEX syndrome. He et al. studied scurfy (SF) mice, which have a mutation in the Foxp3 gene and a clinical phenotype similar to IPEX syndrome. These mice displayed a significant increase in *Bacteroidetes* and a low abundance of *Lactobacillus* (79), whereas Wu et al. (5) reported that the patients with severe diarrhea had a higher abundance of *Firmicutes* and a lower trend for *Bacteroidetes* than donors (5).

Furthermore, bacterial diversity can be restored by the administration of Foxp3+Treg cells in T-cell deficient mice (80). Similarly, Treg cells were inducted by the gut microbiota (81) or its molecular products, such as the carbohydrate polysaccharide A expressed by the symbiont *B. fragilis* (82) and the *Clostridia*-related segmented filamentous bacteria (83).

#### 2.6.2 Autoimmune polyendocrinopathy–candidiasis–ectodermal-dystrophy

APECED is a rare autoimmune disease characterized by cutaneous candidiasis, hypoparathyroidism, and adrenal insufficiency; two conditions are required for diagnosis (84). Approximately 25% of the patients exhibit gastrointestinal symptoms, including malabsorption, chronic diarrhea, and obstipation (85). Hetemäki et al. analyzed the fecal samples from 15 patients with APECED and identified a reduction in gram-positive *Firmicutes* and an increase in gram-negative *Bacteroidetes* and *Proteobacteria* compared to healthy controls. This finding suggests that these gram-negative bacterial taxa may promote dysbiosis and the inflammatory phenotype *via* biofilm production and increased exposure to LPS (86).

#### 2.6.3 IL-10 receptor deficiency

IL-10 inhibits both adaptive and innate pro-inflammatory immune responses and promotes maintenance of mucosal homeostasis (87). Mutations in IL-10 and its receptors (IL-10R) can lead to early onset and severe IBD (88). Several studies have employed IL-10 deficient mice to establish the relationship between the microbiome and colitis disease penetrance. However, the information available on the human microbiome is limited. By exploring the fecal microbiome composition of patients with loss-of-function mutations in the *IL10RA* gene, Xue et al. reported a decreased diversity of the gut microbiome with increased variability and the predominance of *Firmicutes*, *Proteobacteria*, *Actinobacteria*, and *Bacteroidetes*. Furthermore, gut dysbiosis exhibited a moderate association with disease severity (89).

### 2.7 IEI with defects in phagocytes

Being the essential elements of innate immunity, phagocytes (polymorphonuclear leukocytes, macrophages, and dendritic cells) internalize and destroy pathogens and trigger the adaptive response through antigen presentation (90). Intestinal phagocytes play an important role in the maintenance of intestinal homeostasis, including immune tolerance to symbionts and immune recognition of pathobiont. Thus, gut microbiota dysbiosis could lead to the impairment of the innate immune system, especially phagocytic cells (91).

#### 2.7.1 Chronic granulomatous disease

Chronic granulomatous disease (CGD) is an oxidative burst defect resulting from a genetic defect in the nicotinamide dinucleotide phosphate (NADPH) oxidase (NOX) complex subunits and is associated with recurrent life-threatening bacterial and fungal infections and development of abnormal granulomas in tissues (92). These granulomas can affect several organs, particularly the gastrointestinal tract and the brain, lungs, liver, spleen, eyes, and genito-urinary tract (18). Gastrointestinal disorders are a common initial symptom of CGD and precedes the diagnosis in up to 17% of patients (93). Patients with CGD can develop intestinal inflammation mimicking IBD, especially those with p40phox deficiency (94). Through a characterization of fecal microbiota composition derived from 11 patients with CGD, 7 patients with an X-linked inhibitor of apoptosis (XIAP) deficiency, and 7 patients with partial tetratricopeptide repeat domain 7A (TTC7A, 7 samples), Sokolo et al. (95) observed a significant increase in *Proteobacteria* of the Enterobacteriaceae family in the TTC7A group and an increase in bacteria of the *Bacteroidetes* phylum and the Clostridiaceae family in the CGD and XIAP groups, respectively. Interestingly, in patients with XIAP, bacteria that are normally part of the oral microbiota were identified, suggesting a direct effect of these oral bacteria on the gut inflammatory phenotype observed in patients with XIAP deficiency (95). Another study demonstrated that oral microbiota may result in negative health outcomes, including IBD, when they colonize the gut (96).

#### 2.7.2 Kostmann disease

Kostmann disease is an autosomal recessive severe congenital neutropenia caused by mutations in HCLS1-associated protein X-1 (HAX1) with aberrant cell death of the myeloid progenitor cells (97). Patients with Kostmann syndrome suffer from life-threatening bacterial infections such as omphalitis, skin infections, otitis, tonsillitis, abscesses, and sepsis (98). Kostmann disease can also manifest *via* cognitive and neurological abnormalities in patients with deficiency in both HAX1 isoforms, with the increased risk of Myelodysplastic syndrome/leukemia (2). Additionally, this severe congenital neutropenia can affect periodontal neutrophils, influencing the host–microbe homeostasis and leading to early-onset periodontal breakdown (99). Topcuoglu et al. explored the oral microbiota of a group of nine Kostmann disease cases and reported a decreased bacterial diversity with a higher proportion of *Firmicutes* in patients with Kostmann disease compared to the healthy controls, while *Proteobacteria* were more abundant in the control group (100). To elucidate the immunological mechanisms of susceptibility to oral microbial dysbiosis in patients with severe congenital neutropenia (SCN), Zaura et al. investigated the oral microbiome in 10 cases of severe congenital neutropenia and observed markedly higher levels of cytokines and high bacterial load with low bacterial diversity in the saliva of patients with SCN (101). These findings suggest that disrupted immune response to the oral microbiota might be involved in the observed clinical and microbiological manifestations.

## 3 Interplay between skin microbiome and IEI

The skin is the largest organ of the human body, measuring an average of 30 m^2^ (102). The microbiome of a healthy human skin exhibits significant topographical variation between sebaceous, dry, and moist microenvironments and is dominated by *Actinobacteria*, *Bacteroidetes*, *Cyanobacteria*, *Firmicutes*, and *Proteobacteria.* Viruses represent a small part of the cutaneous microbiota of a healthy subject and are essentially bacteriophages (103). IEIs with mucocutaneous manifestations have been chosen to assess the relationship between skin microbiota and disease progression during IEI. Oh et al. studied the bacterial and fungal microbiomes of the skin in patients with WAS and hyper-IgE (STAT3-deficient) and cytokinesis 8 (Dock 8) syndromes. They noted increased permissiveness and decreased colonization by microbial species that were not observed in controls, particularly *Clostridium* spp. and *Serratia marcescens*, with an increased presence of opportunistic fungi (e.g., *Candida, Aspergillus*), thereby supporting the increased permissiveness of the skin for IEI (104). Smeekens et al. compared oral and skin microbiomes of patients with HIES and autosomal dominant chronic mucocutaneous candidiasis (CMC) caused by mutations in STAT3 and STAT1, respectively. Both of these mutations decrease Th1 and Th17 responses, resulting in a higher susceptibility to infection by *Staphylococcus* and *Candida* species (105). Furthermore, the skin microbiome of patients with CMC and HIES was characterized with the abundant presence of *Acinetobacter* and reduced presence of normal *Corynebacterium* compared to healthy controls. The exposure of human primary leukocytes to *Acinetobacter* inhibited the cytokine response to *C. albicans* and *S. aureus*, thereby contributing to the increased risk of infections, whereas normal *Corynebacteria* did not suppress cytokine responses (105). In contrast, Tirosh et al. investigated the viral composition of the skin microbiota of patients with DOCK-8 deficiency and demonstrated enhanced eukaryotic viral representation and diversity compared to healthy volunteers (103). DOCK8 deficiency is a rare autosomal recessive combined immunodeficiency characterized by severe eczema, recurrent cutaneous and systemic infections, cancer susceptibility, and allergic manifestation (106). DOCK8 encoding a guanine nucleotide exchange factor strongly present in lymphocytes has an anti-viral effect by regulating the actin cytoskeleton, which is essential for lymphocyte migration through compact collagenous tissues (107). The importance of its role is illustrated by the severity of skin infections associated with DOCK-8 deficiency, including molluscum contagiosum, herpes virus infections, and HPV infections.

## 4 Interplay between lung microbiome and IEI

Previously considered sterile (108), the respiratory tract, as well as gut and oral localizations, also contains a complex community of microbiota (8) characterized by a relative dynamic due to the microbiome elimination *via* aspiration, cough, or mucociliary clearance (109). The microbiota of the lungs is more similar to the oral microbiome than other localizations (110). The most abundant phyla of healthy lung microbiomes are *Firmicutes* and *Bacteroidetes*; major genera include *Prevotella*, *Veillonella*, and *Streptococcus* (108). Furthermore, interactions between lung immune cells and local microbiota are important in maintaining an immune tolerance toward these commensal bacteria (111). Each inflammatory pathology of the respiratory tract responsible for the production of intra-alveolar catecholamines and inflammatory cytokines results in a change in microbial growth conditions, thereby favoring the growth of certain bacterial species (e.g., *P. aeruginosa*, *S. pneumoniae*, *Staphylococcus aureus*, and *Burkholderiacepacia* complex) (108, 112, 113). Several IEIs (e.g., CVID, sIgAD, IPEX, HIES, and DiGeorge syndrome) are associated with respiratory disorders, including increased susceptibility to respiratory tract infections and asthma development (114). By investigating immunodeficient Rag1-/- mice, Nunzi et al. reported that lung microbiota was able to substitute the lack of B and T-cells by increasing the abundance of beneficial anaerobes such as *Bacteroidota* and *Clostridia*. In contrast, NSG (NOD scid gamma) mice, which have severe defects in the innate and adaptive immune responses, exhibited increased susceptibility to infections and were associated with reduced abundance of strict anaerobes and expansion of *Proteobacteria* (115). The characteristics of the main IEI associated with dysbiosis are summarized in **Table 1**.

**Table 1:** Dysbiosis categories and changes associated with IEI clinical phenotypes.

Type of Dysbiosis	IEI phenotype	Clinical characteristics	Microbiota changes	
			Increase	Decrease
Gut	SCID (28, 30–32)	Early onset of severe infections Early death in the absence of early management.	*Escherichia*, *Staphylococcus Enterococcus* *Streptococcus* *Astrovirus, Bocavirus, and Adenovirus.*	–
	WAS (33, 38)	Eczema, recurrent infections, autoimmunity and malignancies.	*Roteobacteria*	*Bacteroidetes* *Verrucomicrobia*
	CVID (41, 57)	Recurrent infections specially in the respiratory tract.	*Bacilli* *Gammaproteobacteria* *Clostridia* *Prevotella*	*Actinobacteria (Bifidobacteriaceae)* *Faecalibacterium)* *Bacteroides* *Firmicutes*
	SIgAD (2, 18, 116)	May be asymptomatic. Bacterial infections, autoimmunity mildly increased.	*Firmicutes Bacteroidetes Gammaproteobacteria Prevotella*	*Firmicutes (Lachnospiraceae* and *Faecalibacterium) Bacteroides*
	HIES (2, 68)	Distinctive facial features (broad nasal bridge) Bacterial infections (boils and pulmonary abscesses, pneumatoceles) due to *S. aureus*, *Aspergillus, Pneumocystis jirovecii*; Eczema; Mucocutaneous candidiasis; Hyperextensible joints, osteoporosis and bone fractures, scoliosis, retention of primary teeth; Aneurysm formation.	*Candida albicans*	Minimal representation of health-associated fungi (*C. parapsilosis, Boletus* and *Penicillium species)*
	IPEX (5)	Autoimmune enteropathy, Early onset diabetes, thyroiditis hemolytic anemia, thrombocytopenia, eczema, elevated IgE and IgA.	*Firmicutes*	*Bacteroidetes*
	APECED (84, 86)	Autoimmune disease characterized by cutaneous candidiasis, hypoparathyroidism and adrenal insufficiency, two of which are required for diagnosis.	Gram-negative *Bacteroidetes* and *Proteobacteria*	Gram-positive *Firmicutes*
	IL10RA (89)	Folliculitis, recurrent respiratory diseases, arthritis, lymphoma.	*Firmicutes, Enterococcaceae, Enterococcus, Lactobacillales*, *Bacilli*, *Micrococcales*	*Bifidobacteriales, Bifidobacteriaceae, Bifidobacterium, Veillonellaceae, Clostridiales, Clostridia, Selenomonadales, Negativicutes*
	CGD (92, 95)	Recurrent life-threatening bacterial and fungal infections and abnormal tissue granuloma formation	*Ruminococcusgnavus*,	–
	XIAP (2, 95)	Splenomegaly, lymphoproliferation, Colitis, IBD, hepatitis.	*Proteobacteria*, *Firmicutes* *Actinobacteria* *Fusobacteria*	–
	TTCA7TTC7A deficiency (95, 117)	Mutations in this gene usually result in gastrointestinal defects and immunodeficiency.	*Proteobacteria Proteobacteria*	*Ruminococcaceae* (*Oscillospira*)
Oral	WAS (39)	Oral involvement: gingivitis and periodontitis; Premature loss of deciduous and permanent teeth.	*Fusobacterium nucleatum Porphyromonas gingivalis* *Tannerella forsythia*	–
	Kostmann (2, 101)	AR. Cognitive and neurological defects in patents with defects in both HAX1 isoforms, susceptibility to MDS/leukemia	*Firmicutes*	–
Skin (18, 104, 105)	AD-HIES	–	*Acinetobacter*	*Corynebacterium*
	STAT3-HIES	–	*Serratia MarcescencesppAnaerococcus, FinegoldiaandPeptoniphilus* *S. Epidermidis spp opportunistic fungi (Candida, Aspergillus)*	*Porphyromonas* *Cloacibacterium Propionibacterium*
	WAS	–	*Propionibacterium*	–
Lung	RAG1-/- mice (115)	–	Abundance of beneficial anaerobes: *Clostridia* and *Bacteroidota.*	–
	NSG (NOD SCID gamma) mice (115)	–	*Proteobacteria*	Low abundance of strict anaerobes

SCID, severe combined immunodeficiency; WAS, Wiskott-Aldrich syndrome; CVID, common variable immunodeficiency; sIgAD, selective IgA deficiency; HIES, hyper IgE syndrome; IPEX, immune dysregulation, polyendocrinopathy, enteropathy, X-linked; APECED, Autoimmune polyendocrinopathy-candidiasis-ectodermal-distrophy; IL10RA, interleukin-10 receptor subunit alpha; CGD, chronic granulomatous disease; XIAP, X-linked inhibitor of apoptosis; TTC7A, Tetratricopeptide Repeat Domain 7A;AD-HIES, Autosomal dominant hyper-IgE syndrome; STAT3-HIES, STAT3 hyper-IgE syndrome.

## 5 Can microbiome analysis contribute to IEI diagnostic strategy?

### 5.1 Diagnostic strategy of IEI

When immune deficiency is suspected and a patient’s family history analysis and a clinical examination have been conducted, the types of complications observed and the presence of syndromic signs associated with simple first-line biological examinations (e.g., HIV serology, complete blood count, immunoglobulin and/or protein electrophoresis assay, complement assay, and post-vaccination serology) make it possible to immediately evoke well-defined primary immunodeficiency entities requiring a specific etiological assessment as second-line biological examinations (e.g., subpopulation phenotyping, lymphocyte proliferation test, oxidative burst test, analysis of the expression of a specific membrane or intracytoplasmic protein) (118). First- and second-line examinations are often supplemented by genetic analysis, which enables confirmation of the suspected molecular defect (119).

For some IEIs with dysbiosis, including CIVD (21, 42), WAS (38), and associations with gastrointestinal expression, the different manifestations may represent a particular interest when identifying potential associated microbiomes. Despite a routine evaluation of a patient’s microbiome for diagnostic purposes not currently being recommended for IEI, some studies consider that gut microbiome analysis can help identify an underlying immunodeficiency in patients with IBD (37), and the alteration in the gut microbiota composition might be of clinical interest as diagnosis biomarkers (95). Moreover, as an important contributor to the individual immune cell function, clinical phenotype, and therapeutic response, microbiomes can be assessed in conjunction with other methods of immune cell evaluation in the context of IEI (120).

### 5.2 Microbiome identification

In addition to routine microbiology testing allowing for the detection of single microbes such as bacteria, viruses, and fungi, which are isolated from patients with acute or chronic infections, nowadays, emerging molecular analysis based on genomics, transcriptomics, proteomics, and metabolomics allows for the detection and characterization of various microorganisms in the gastrointestinal tract, skin, lungs, and urogenital tract, among others. These advances in biotechnology offer the possibility of assessing all genomes and gene products of a microbiome (121).

Microbiology has always heavily relied on cultures, and bacterial culture was performed in early studies of the human microbiome. Owing to the difficulty in culturing numerous skin bacteria, only a limited amount of information on the skin microbiome was known prior to the development of NGS technologies. In the current field of skin microbiome research, two methodologies are most commonly used. The first is amplicon sequencing, which relies on sequencing taxonomic marker genes (usually 16S ribosomal RNA (rRNA) gene for bacteria and archaea and internal transcribed spacer (ITS) gene for fungi) after initial targeted PCR amplification and works effectively for samples contaminated with host DNA, such as tissue and low-biomass samples (122, 123). The second is metagenomic shotgun sequencing, which collects all genetic information simultaneously (123). New microbiota identification techniques can reveal important host–microbe interactions, such as precisely identifying and quantifying commensal microbiota bound to host immunoglobulins. Immunoglobulin binding of commensal taxa can be determined by sorting the bound bacteria from samples (flow cytometry) and determining their taxonomy *via* amplicon sequencing, a technique most commonly used to study IgA (IgA-Seq) (124).

## 6 Microbiome-based therapeutic strategies in IEI

As the relationship between the gut microbiota, host immunity, and infectious diseases becomes better understood, the integration of microbiota modulation as a strategy of therapy or infection prevention into daily clinical practice becomes necessary. This is particularly prevalent as gut microbiota alteration is not only a consequence but also actively involved in the inflammatory process (125). Researchers are developing optimized therapies and therapeutics such as prebiotics, probiotics, postbiotics, and FMT to rebalance the gut microbiota and attenuate disease activity in many IEIs. FMT involves placing the stool from a healthy donor into the digestive tract of a recipient patient to restore the disrupted gut microbiome, thereby providing a therapeutic benefit (126, 127). Several studies have demonstrated that FMT is a treatment available for recurrent and/or refractory *Clostridium difficile* infections (CDI) even in immunocompromised patients (128–130). Patients with IEI have been included in four studies (131–134), two of which reported a lack of safety concerns and successful resolution of CDI (133, 134). Wu et al. investigated the effect of FMT before HSCT in a child with IPEX and reported a remission of diarrhea without significant side effects with increased microbial diversity as well as a change in the microbiota composition. However, complete cure of the disease was only obtained after HSCT (5). Probiotics are naturally live microorganisms that are orally administered to treat dysbiosis (135). Their mechanisms of action are based on increasing the production of antimicrobial peptides; maintaining the integrity of the gastrointestinal epithelial barrier; and optimizing the interactions between gut microbiota, intestinal epithelial cells, and mucosal immune cells (136). Liu et al. showed that in the mouse model of IPEX syndrome, gut microbial dysbiosis could be corrected using the human probiotic *Lactobacillus reuteri* DSM 17938, which is a human-derived probiotic that has been used in the treatment of infantile colic and acute infectious diarrhea (71). Similarly, He et al. demonstrated that remodeling of the microbiota with *L. reuteri* in SF mice protects them against Treg cell deficiency-induced autoimmunity by suppressing Th1/Th2 cells *via* inosine–adenosine A2A interaction (79).

Previous studies using mouse models with innate immune deficiency have reported high serum IgG levels directed against gut microbiota (137). Similarly, significant titers of IgG targeting *Escherichia coli* have been reported in patients with IBD and in secretory IgA-lacking mice (138, 139), while recent murine studies have shown that under homeostatic conditions, healthy mice actively produce systemic IgG against commensal bacteria (140). These serum IgGs provide systemic protection against commensal flora and are directed against conserved motifs of the flora (141). Fadlallah et al. confirmed the presence of human anti-commensal IgG directed against a broad spectrum of commensal bacteria, and reported that under homeostatic conditions, secretory IgA and systemic IgG bind to a common spectrum of commensals (116). This finding provides new therapeutic perspectives in IgA-deficient patients based on intravenous immunoglobulin supplementation with IgG from IgA-deficient patient pools to offer better protection against gut bacterial translocations in patients with CVID (116). Mohammed et al. focused on the effect of gluten-free diet (GFD) on microbiota in B cell-deficient mice and found that it suppressed the expansion of anaerobic bacteria in the small intestine and colonization of the small intestine by a specific pathobiont (142).

Furthermore, other studies suggested metabolic byproducts from gut microbiota (e.g., SCFAs) could cause epigenetic modification by affecting the local homeostasis of phagocytes and myelopoiesis in the bone marrow. The major mechanism for SCFAs is based on the direct inhibition of histone deacetylases (HDACs) and activation of G-protein-coupled receptors (GPCRs) (143).

Recently, Falcone et al. reported that exclusive enteral nutrition over 10 weeks induced sustained changes in the microbiota and improved IBD in a pediatric patient with an X-linked chronic granulomatous disease (144). Furthermore, the patient’s gut microbiome was markedly enriched with *Faecalibacterium prausnitzii* (144), a bacterium with anti-inflammatory properties that is associated with healthy gut microbiota (145).

## 7 Conclusion

The interplay between microbiota and host immunity is a fundamental, symbiotic, and dynamic relationship. In genetically predisposed individuals, microbiome dysbiosis can amplify the defective immune response against microbial and fungal pathogens. In turn, the microbiota can be shaped by the immune system, for instance, *via* the release of antimicrobial peptides or IgA. In the particular context of IEIs, dysbiosis may occur because of a decrease in the population of microorganisms that are normally associated with a protective effect on the host, an increase in pathogen population, or an ectopic localization of the commensal flora. In parallel to the number of IEIs currently characterized in patients, additional information is also emerging. Therefore, larger and specific studies focusing on the molecular, immunological, and clinical aspects are required to better understand IEIs and related microbiome interactions. Moreover, with the gradual understanding of microbiota, the potential of treating diseases by manipulating microbiota is becoming more achievable, and therefore, further investigation of the microbiota and their products may facilitate the development of novel therapeutic strategies for patients with IEIs.

## Author contributions

RH analyzed literature and wrote the manuscript. BA designed, supervised the project and critically reviewed thefinal draft. FE contributed to the drafting of the manuscript. AAB contibuted to the design and validation of the clinical aspect of the manuscript. All authors contributed to the article and approved the submitted version.

## Acknowledgments

*We apologize to those investigators whose work we were unable to cite for space limitations.*

## Conflict of interest

The authors declare that the research was conducted in the absence of any commercial or financial relationships that could be construed as a potential conflict of interest.

## Publisher’s note

All claims expressed in this article are solely those of the authors and do not necessarily represent those of their affiliated organizations, or those of the publisher, the editors and the reviewers. Any product that may be evaluated in this article, or claim that may be made by its manufacturer, is not guaranteed or endorsed by the publisher.
